# DNA Methylation Dynamics in *Eisenia andrei* Regeneration: The Effects of Time, Region, and a Hypomethylating Agent

**DOI:** 10.3390/biom16081192

**Published:** 2026-08-14

**Authors:** Chayeen Brotzki da Costa, Péter Németh, Péter Engelmann

**Affiliations:** Department of Immunology and Biotechnology, Medical School, Clinical Center, University of Pécs, 7624 Pécs, Hungary; brotzki.chayeen@pte.hu (C.B.d.C.); nemeth.peter@pte.hu (P.N.)

**Keywords:** DNA methylation, decitabine, epigenetics, earthworm, regeneration

## Abstract

The impact of epigenetic mechanisms on molecular and cellular processes of regeneration remains a less-investigated field, especially concerning earthworm segment restoration. To evaluate distinct methylated cytosines under different conditions, earthworm segments were collected: decitabine-treated and controls; anterior and posterior amputation; intact and regenerated (2- and 4-week). 5-methylcytosine (5mC) and 5-hydroxymethylcytosine (5hmC) distribution in tissues was assessed by immunohistochemistry (IHC), and in genomic DNA by dot blot. DNA-methyltransferase (DNMT) and ten-eleven translocation (TET) dioxygenase activity was determined by immuno-based colorimetry. *DNMT1* and *TET* gene expressions were verified with real-time PCR. Decitabine treatment reduced 5mC levels in most tissues, persisting in the anterior coelomic cavity (intact and blastemas). 5hmC remained elevated in most tissues, remarkably in 2-week blastemas. In the same period, DNMT and TET presented the highest activity in anterior treated samples compared to other treated periods. Concurrently, *DNMT1* gene expression increased prominently in anterior segments, while *TET* showed the highest expression in both anterior and posterior 2-week treated groups. Thus far, our observations indicate that, despite decitabine’s effect on the DNA methylation machinery of the earthworm model, the region of amputation and the regeneration period also interfere with activity and expression of epigenetic enzymes, affecting 5mC and 5hmC distribution.

## 1. Introduction

Several pathways described in regeneration combine steps of normal embryonic and post-embryonic development, which strongly rely on epigenetic regulation [1,2,3]. Attenuation of stem cell potential and dynamic interactions with gene silencing are essential components in the establishment of cell fate decision, which is also shaped by epigenetic mechanisms [4,5]. Based on this understanding, the impact of the epigenome on normal development through the control of gene expression can be reflected in a possible interaction with regeneration as well [6,7]. Described in vertebrates and invertebrates, the ability to restore lost or injured body parts and organs is carried out through morphallaxis or epimorphosis [8,9,10]. Morphallactic regeneration is characterized as the spatial reorganization of cells in the remnant tissue, while the epimorphic process involves the proliferation of undifferentiated cells to form a blastema [10]. Both mechanisms are present in various animals, and it is assumed that they occur simultaneously, in a balance necessary to establish tissue homeostasis and reconstruction of structures [9,10].

Despite the conserved character of the molecular components involved in building and maintaining epigenetic tags throughout the Tree of Life, notable differences in frequency and type of epigenetic mechanisms can be observed among taxa [11,12]. Amidst the multiple forms of epigenetic modifications, DNA methylation certainly stands out for being widely detected across the animal kingdom, and its functions are extensively characterized in a multitude of processes [4,5,6,13]. The so-called “fifth base”, 5-methylcytosine (5mC), is a key epigenetic modification that has multiple functions through gene expression regulation, including the maintenance of cell identity and control of lineage specification during development [14,15].

The generation of methylation tags is performed by DNA methyltransferase (DNMT) enzymes, mainly DNMT1 and DNMT3, using a methyl group from S-adenosylmethionine (SAM) [16]. DNMT1 is responsible for the maintenance of DNA methylation in CpG islands, ensuring that the methylation pattern established in a cell is faithfully copied to the newly synthesized DNA during replication [17,18], while DNMT3a and DNMT3b are responsible for de novo methylation [19]. This modification of the epigenetic landscape is mainly associated with development and cellular differentiation, correlating with tissue-specific gene expression profiles [20,21].

Additionally, the regulation of DNA methylation is featured by ten-eleven translocation proteins (TET dioxygenases) [21]. This family of enzymes takes part in the active demethylation process, characterized by the enzymatic removal of methyl groups from cytosine nucleotides [22]. A product of 5mC oxidation, 5-hydroxymethylcytosine (5hmC), is formed by TET dioxygenases, which catalyze the conversion of 5mC into 5hmC. This variation in methylated cytosine appears enhanced during mouse embryonic development, for example, being associated with active gene expression and tissue plasticity, easing gene expression regulation during differentiation and tissue repair [23,24]. Many stages of development in different organisms count on the action of TET dioxygenases to reduce global DNA methylation states and modulate stem cell differentiation and tissue reprogramming [22,25]. Alternatively, passive demethylation occurs when DNA methylation is not maintained during the replication process, leading to a gradual reduction of DNA methylation tags over time. In a scenario where maintenance DNMT is not active, cell proliferation results in a diluted state of methylation marks, which are subsequently lost through further cell divisions [21,24].

The influence of the epigenome on molecular and cellular processes of regeneration remains an underexplored area, particularly in annelids and, more specifically, in earthworm segment restoration. Like other members of the Oligochaeta subclass, many earthworm species have a great capacity to restore lost or injured segments, even in both anterior and posterior regions, as is the case for *Eisenia andrei*. This remarkable ability is reported in adult animals (after reaching sexual maturation), which also correlates with a possible function of the clitellum in regenerative processes [26]. Anatomical features of annelids, such as the presence of a coelomic cavity and a body organized in segments, might also assist during wound healing and tissue restoration [27]. Most model organisms used in regenerative studies present well-defined stages of tissue repair, as is the case with the axolotl, for example: upon injury, wound healing and a bud cap formation occur, followed by the formation of a blastema that resembles the embryonic limb in structure and function, and later it differentiates into the lost limb [28]. The segment regeneration in *E. andrei* follows a similar kinetics, where the hallmarks are also composed of wound healing and plug formation up to a few hours after injury or amputation. Subsequently, a significant increase in cell proliferation leads to the formation of a mass of undifferentiated cells—the blastema—at the time point corresponding to 2 weeks post-amputation. After 4 weeks of the initial stress and following several cellular and molecular events, the blastema differentiates, with the return of skin pigmentation, development of segment appendages, and structure and function that resemble the original lost tissue [9,10,29].

Moreover, several components of the innate immune system also take part in events contributing to the tissue regeneration of earthworms [30]. In a recent study of how innate immunity influences the regeneration process in *E. andrei*, we observed biased transcripts of several immune response-related genes (pattern recognition molecules and antimicrobial factors) during earthworm regeneration. Furthermore, the depletion of cellular immune components (e.g., coelomocytes) impaired differentiation and cell proliferation in both posterior and anterior blastema during segment restoration in earthworms [31].

Hypomethylating agents (HMAs) have been applied for many years in the treatment of malignancies and other conditions, reflecting the role of DNA methylation in pathogenesis and disease progression. Decitabine (5-aza-2′-deoxycytidine, 5-Aza) is a well-known drug that presents hypomethylating effects at low doses and is employed in the treatment of myelodysplastic syndrome and leukemias [32]. Furthermore, this cytosine analog has been described as modulating appendage regeneration in axolotl and osteocyte differentiation in vitro [33]. By physically inhibiting DNMT1 from maintaining 5mC tags during replication, it modulates gene expression regulation and is also thought to interfere with other components of the DNA methylation machinery [34]. Earlier, decitabine has been applied in toxicological and regeneration studies in different annelid models [12,35].

Given the wide variation in regenerative capacity across species and the well-established role of epigenetic mechanisms in shaping developmental processes, this study aims to investigate the occurrence of different cytosine modifications (5mC and 5hmC) in earthworm regeneration. More specifically, we evaluate their distribution in distinct tissues and body regions during different regeneration stages, as well as under chemical modulation. By assessing the dynamics and possible impact of such factors in earthworm segment restoration, we seek to clarify how epigenetic mechanisms contribute to regeneration.

## 2. Materials and Methods

### 2.1. Experimental Groups and Sample Collection

Adult clitellate *E. andrei* earthworms (Oligochaeta, Lumbricidae) were kept at room temperature and fed with manure soil. The specimens had a mean body weight of 0.3 g and length of 5.6 cm. One day before amputation or exposure, the animals were transferred to containers with moist paper towels for gut depuration. Animals were anesthetized in carbonated water for approximately 60 s, then five segments were amputated from either the anterior or posterior regions. Intact segments were collected at the time of amputation, while blastemas (restored segments) were collected 2 and 4 weeks after amputation. This temporal framework was determined based on a similar study [36], facilitating comparisons [37,38]. Earthworms were maintained in soil at room temperature throughout the regeneration period. For an illustrative explanation of the experimental groups, amputation, and temporal framework, please consult the scheme provided in the Supplementary Materials (Figure S1).

### 2.2. Exposure to Hypomethylating Drug

Adult earthworms were exposed to a 5 µM solution of 5-aza-2′-deoxycytidine (Decitabine, Sigma-Aldrich A3656, Darmstadt, Germany) diluted in LBSS buffer (*Lumbricus* Balanced Salt Solution) [39] for 48 h through filter paper. Control groups were exposed to LBSS only. Intact and regenerated segments were collected as previously described. The working concentration of Decitabine and exposure method were determined after trial using different concentrations (1, 2, and 5 µM) in different media (liquid, filter paper, and soil), and later assessment of 5mC levels by immunohistochemistry and dot blot assay, together with evaluation of the mortality rate of the animals.

### 2.3. Immunohistochemistry

Segments collected from each experimental group were embedded in Killik^®^ O.C.T cryostat embedding medium (Bio-Optica, Milan, Italy). Sections (8 micron-thick) were fixed in 4% paraformaldehyde (PFA) and subjected to antigen retrieval in heated sodium citrate solution (0.01 M, pH 6.0) for 10 min. Endogenous peroxidases were inhibited by incubation with phenylhydrazine hydrochloride (1 mg/mL, Sigma-Aldrich, Darmstadt, Germany), and unspecific binding sites were blocked with 5% bovine serum albumin (BSA) solution. Slides were incubated for 2 h with anti-5mC (mouse IgG, clone 33D3, Merck, Darmstadt, Germany) or anti-5hmC (mouse IgG, clone HMC 31, Merck, Darmstadt, Germany) monoclonal antibodies (mAbs) at a 1:100 dilution ratio. Two negative controls were applied: one by omitting the primary antibody and another using an in-house-developed anti-FITC (mouse IgG) mAb, also in a 1:100 dilution. An HRP-conjugated polyclonal rabbit anti-mouse immunoglobulin (Agilent Dako, Glostrup, Denmark) was used as the secondary antibody for 1 h at a 1:100 dilution. Signal was developed using H_2_O_2_/Tris-HCl buffer as substrate and 3,3′-diaminobenzidine (DAB) as chromogen (Histols^®^-DAB, Histopathology, Pécs, Hungary). Microscopy was performed with an Olympus BX-61 microscope, with brightfield images being acquired under the same exposure conditions. Zeiss Zen Blue software (version 2.1, Zeiss, Jena, Germany) was used for image analysis.

### 2.4. Genomic DNA Isolation and Dot Blot Assay

Intact and regenerated tissue samples from control and exposed animals were used for genomic DNA isolation with NucleoSpin^®^ Tissue kit (Macherey-Nagel, Düren, Germany) following the manufacturer’s protocol (with lower elution buffer volume). For each experimental group, segments/blastemas from 5 animals were collected (*n* = 5). DNA concentration and quality were assessed by NanoDrop^®^ (Thermo Fisher Scientific, Waltham, MA, USA). Genomic DNA samples were diluted (to 10 ng/µL) and denatured (1% 4 M NaOH solution, 42 °C, 12 min).

Samples were spotted in triplicate (3 experimental/technical replicates) onto a nitrocellulose membrane (10600001 GE Healthcare, Chicago, IL, USA); unmethylated CpG DNA (HC4039, Hycult Biotech, Wayne, PA, USA) at a concentration of 10 ng/µL was used as a negative control. After air-drying and blocking (3% BSA), the membrane was incubated (overnight, 4 °C, under rotation) with the primary antibodies described earlier at a 1:5000 dilution ratio in blocking solution. A polyclonal rabbit anti-mouse HRP-conjugated immunoglobulin (Agilent Dako, Glostrup, Denmark) was used at a 1:2000 dilution ratio as the secondary antibody. Signal was developed with chemiluminescent substrate SuperSignal^®^ West Pico PLUS (Thermo Fisher Scientific, Waltham, MA, USA) and registered with ChemiDoc^®^ Imaging System (Bio-Rad Laboratories, Hercules, CA, USA). For further statistical analysis, relative quantification data were obtained with Image Lab software (version 6.0, Bio-Rad, Laboratories, Hercules, CA, USA) using intact control groups as standards.

### 2.5. Nuclear Protein Isolation and Enzymatic Activity Measurement

Intact and regenerated tissue samples from control and exposed animals were submitted to nuclear protein isolation with a nuclear extraction kit (ab113474, Abcam, Cambridge, UK) following the manufacturer’s protocol. Total protein content of the nuclear extracts was determined by BCA assay (Thermo Fisher Scientific, Waltham, MA, USA). For each experimental group, segments/blastemas from 6 animals were collected and processed separately (*n* = 6). Enzymatic activity assays of DNMT and TET dioxygenases were performed using colorimetric kits (ab113467; ab156912, Abcam, Cambridge, UK) following the manufacturer’s instructions. Nuclear extract samples were diluted to the optimal input range (5–10 µg), and experimental triplicates were applied for each sample.

### 2.6. RNA Isolation, cDNA Synthesis, Primer Design, and qPCR

Total RNA was isolated from tissue samples using NucleoZOL reagent (740404, Macherey-Nagel, Düren, Germany). For each experimental group, segments/blastemas from 4 to 6 animals were collected and processed separately (*n* = 4–6). The manufacturer’s protocol was followed after mechanical homogenization of the samples (2.5–5.0 mg) using a Potter homogenizer (Fisherbrand™, Thermo Fisher Scientific, Waltham, MA, USA). Concentration and quality of isolated RNA samples were assessed by NanoDrop^®^ (Thermo Fisher Scientific, Waltham, MA, USA). RNA samples were submitted to DNase I treatment, and cDNA synthesis was performed using the High-Capacity cDNA Reverse Transcription Kit (4374967, Applied Biosystems, Foster City, CA, USA) according to manufacturer’s protocol. Primers were designed to amplify *RPL17* (housekeeping gene), *DNMT1*, and *TET* (target genes) products (please see the Supplementary Materials, Table S1). Available partial cDNA sequences [35,40] were used for multiple alignment with Clustal Omega (version 1.2, University College Dublin, Dublin, Ireland); the most conserved regions within exons were used for primer design with Primer Express (version 3.0, NIH, Bethesda, MD, USA). Quantitative real-time PCR for each target applied Maxima SYBR Green/ROX qPCR master mix (K0222, Thermo Fisher Scientific, Waltham, MA, USA). The experiment was performed using the 7500 Real-Time PCR System (Applied Biosystems, Foster City, CA, USA). The thermal profile consisted of 95 °C for 10 min, followed by 44 cycles of 95 °C for 35 s, 58 °C for 35 s, and 72 °C for 1 min, completed by a dissociation stage of 95 °C for 15 s, 60 °C for 1 min, and 95 °C for 15 s. *RPL17* mRNA level was used for normalization, and gene expression level analysis was performed based on the 2^−ΔΔCT^ method. The independent evaluations were repeated three times in duplicate.

### 2.7. Statistical Analysis

Statistical analysis was performed using Prism 9 (GraphPad Software, version 9.0, Boston, MA, USA). Normality of data distribution was evaluated using the Shapiro-Wilk test (considering alpha = 0.05). For comparisons involving a single variable, one-way ANOVA was applied, while for experiments considering two independent variables, two-way ANOVA was used. When these assumptions were not met, the Kruskal-Wallis test was employed. In each case, Bonferroni multiple comparison test was carried out.

## 3. Results

### 3.1. In Vivo Observations

**Figure 1:**
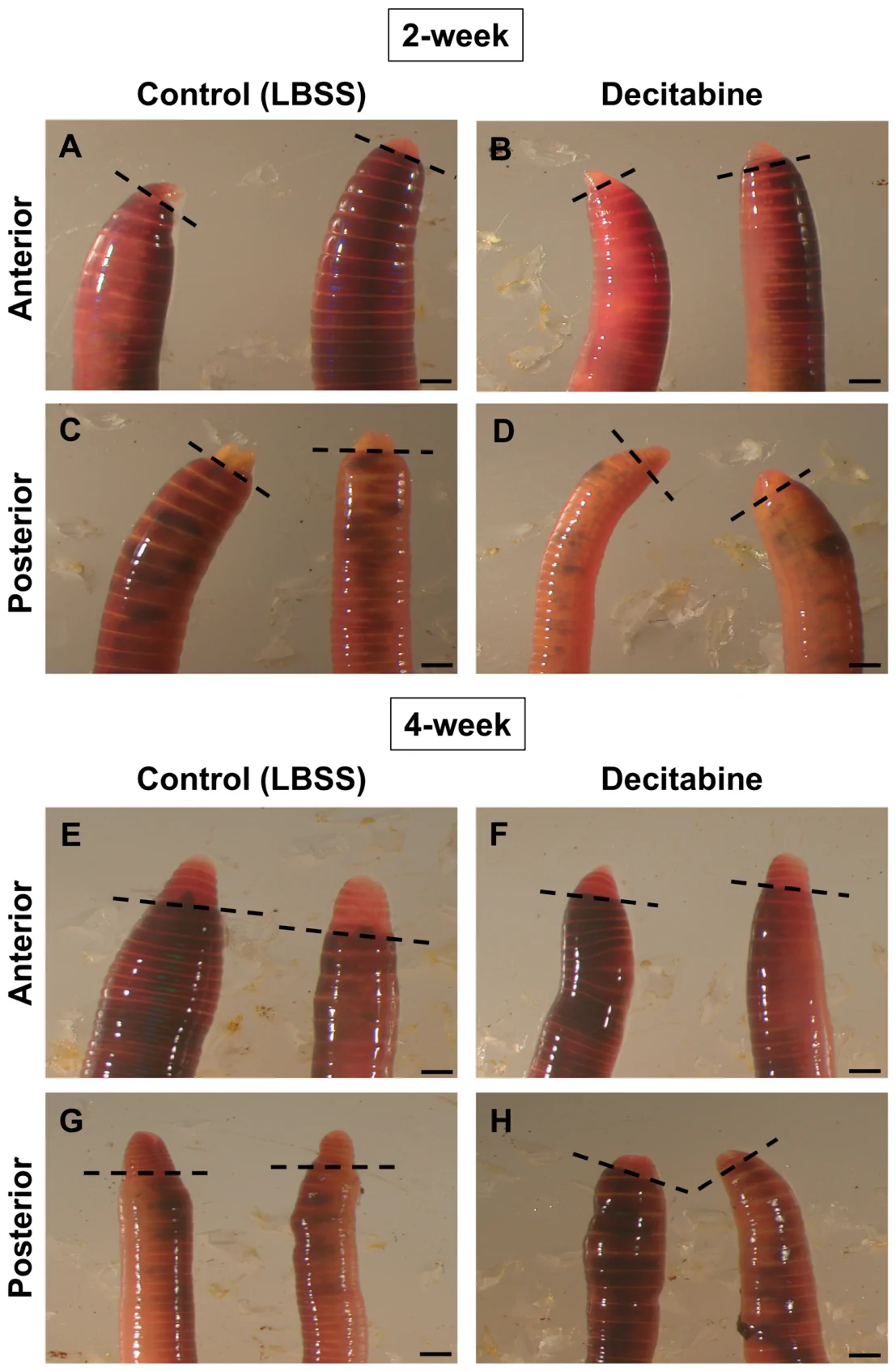
In vivo observations of anterior and posterior regeneration (first and second rows, respectively) of control (**A**,**C**,**E**,**G**) and decitabine (**B**,**D**,**F**,**H**) treated *E. andrei* earthworms at 2 (**A**–**D**) and 4 weeks (**E**–**H**) post-amputation. Dashed lines indicate the separation between the blastema (regenerated tissue) and the original intact segments. Scale bar: 2 mm.

Comparing the treatment groups within regions (anterior or posterior) and periods of regeneration (intact, 2-, and 4-week), no notable anatomical differences were observed. The exposure to decitabine did not alter macroscopically the newly formed segments of the blastema regarding their number, size, pigment, or presence of appendages. However, a slight difference in the number and thickness of segments was observed between regions of amputation. According to our observations, all the decitabine-treated anterior groups—after 2 and 4 weeks of regeneration—presented a mean of 4 new segments, while control anterior groups varied between 3 and 5 new segments in the 2nd and 4th weeks post-amputation, respectively. Posterior regenerated segments were significantly thinner than the anterior ones in both treatments; once again, decitabine-treated groups presented a mean of 4 regenerated segments in both time points, while control animals displayed a mean of 5 and 8 new segments at the 2nd and 4th weeks post-amputation, respectively. In all observations, a total of 6 animals per group were evaluated (Figure 1 and Figure S2) and, according to statistical analysis (Kruskal-Wallis test), the number of newly formed segments does not change significantly between any of the groups.

### 3.2. Tissue Distribution of Global DNA Methylation in Regenerating Earthworms

**Figure 2:**
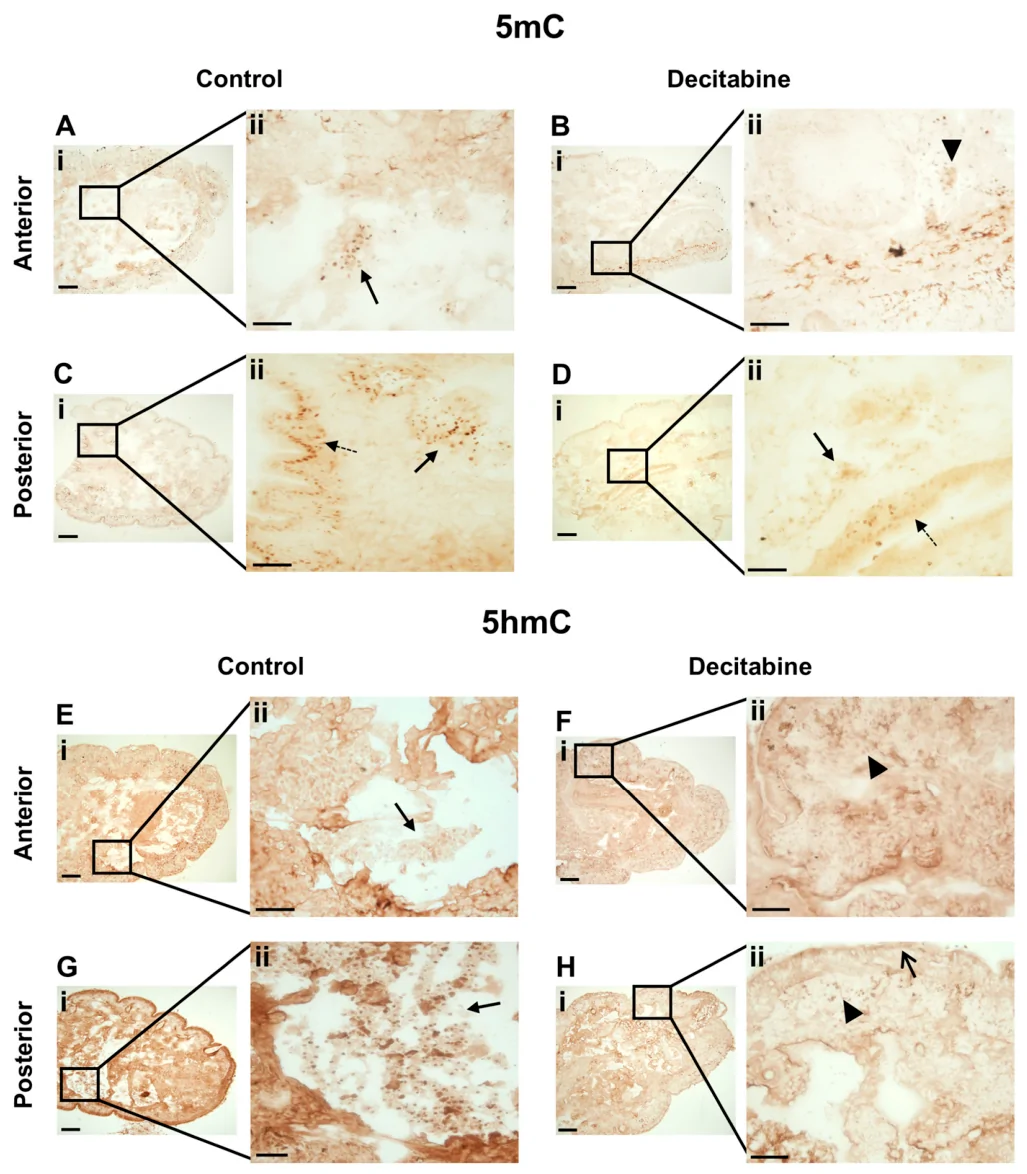
Immunohistochemistry staining targeting 5-methylcytosine (**A**–**D**) and 5-hydroxymethylcytosine (**E**–**H**). (**A**,**E**): control (LBSS) anterior intact, 10× (**i**) and 40× (**ii**); (**B**,**F**): Decitabine anterior intact, 10× (**i**) and 40× (**ii**); (**C**,**G**): control (LBSS) posterior intact, 10× (**i**) and 40× (**ii**); (**D**,**H**): Decitabine posterior intact, 10× (**i**) and 40× (**ii**). Scale bars: 100 µm at 10× magnification; 20 µm at 40× magnification. Target localization is indicated by solid arrows in the coelomic cavity; dashed arrows in the gut; arrowheads in the muscle; line arrows in the skin.

To demonstrate the effect of decitabine exposure, the comparison between treatments immediately after exposure is shown for the intact groups in both the anterior and posterior regions in Figure 2. Applying 5mC (Figure 2A–D) and 5hmC (Figure 2E–H) immunohistochemistry, we observed strong and distinguishing staining in *E. andrei* earthworm tissue sections. Higher magnifications (ii) of the images clearly revealed the nuclear localization of 5mC and 5hmC signals (e.g., Figure 2A-ii,G-ii) in various tissues (skin, muscle, coelomic cavity, and gut). Besides, a reduction in the different cytosine modifications can be observed in some of the tissues after decitabine treatment, when compared to the animals exposed to the control buffer LBSS in the same fashion. In the coelomic cavity, free-floating immune cells, so-called coelomocytes, have evidenced positive nuclear signals in the control groups applying the 5mC and 5hmC-specific mAbs (pointed by solid arrows in Figure 2A-ii,G-ii). In the digestive tract of control and decitabine-treated earthworms (indicated by dashed arrows in Figure 2C-ii,D-ii), the subepithelial cell layer facing the gut lumen shows a characteristic strong nuclear localization of 5mC. The persistence of both 5mC and 5hmC is shown mainly in the muscle of decitabine-treated animals (marked by arrowheads in Figure 2B-ii,F-ii,H-ii). On the other hand, 5hmC is more prominent in the skin than 5mC, remaining present after exposure to decitabine (line arrow in Figure 2H-ii). Despite the resemblance between treatment groups during the regeneration stages as observed macroscopically in the in vivo specimens, it is worth illustrating the formation and development of new segments at the different injured sites (Figures S3 and S4).

### 3.3. Fluctuation of Global DNA Methylation Levels in Genomic DNA

**Figure 3:**
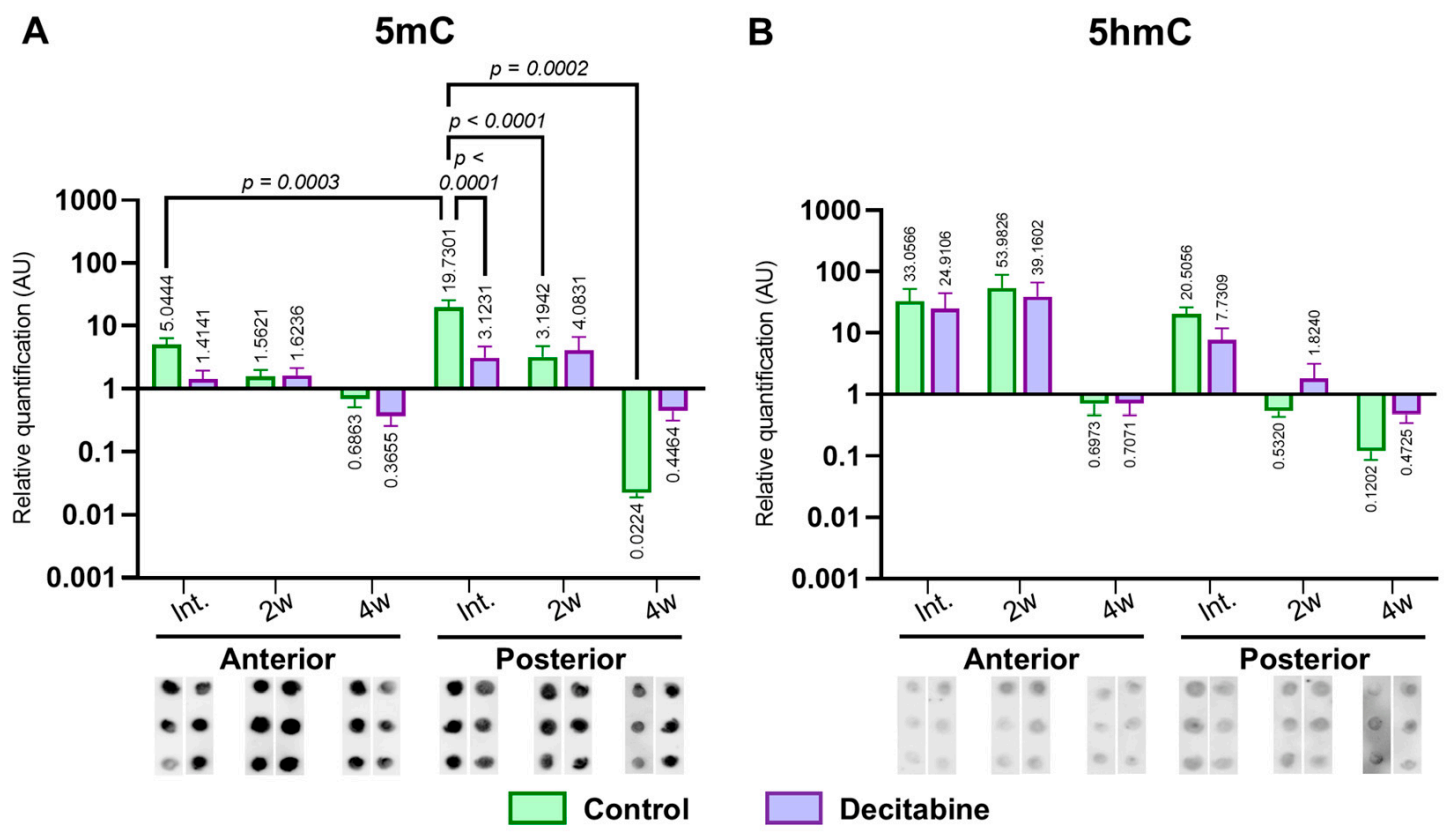
Bar graphs illustrating 5-methylcytosine (**A**) and 5-hydroxymethylcytosine (**B**) levels measured by dot blot assay of intact and regenerated *E. andrei* segments, under treatments (green: control groups [LBSS]; purple: decitabine-treated groups) and at distinct time points. *n* = 5 biological replicates (independent animals), each measured in 3 technical replicates (averaged before analysis). Representative fragments from one biological replicate, spotted in triplicate on the dot blot membranes, are shown under each bar according to the corresponding groups. Mean + SEM; *y*-axis appears on a log_10_ scale for better visualization of the data. AU: arbitrary unit. Comparisons were made between treatments (decitabine vs. control), time points (intact, 2 weeks, and 4 weeks), and regions (anterior vs. posterior).

When comparing treatments, exposure to decitabine was associated with a reduced occurrence of 5mC in the genomic DNA of intact and regenerating earthworms and a significant decrease in the posterior intact group (Figure 3A). This was observed after anterior and posterior segment amputation right after treatment (intact groups), with statistical significance when comparing posterior intact segments of decitabine and control treatments (*p* < 0.0001). 5mC remained reduced in decitabine-treated groups by the 2nd week after anterior and posterior amputation and by the 4th week only in the case of posterior regeneration, in contrast with the corresponding controls. Nevertheless, the relative quantification values presented at the 4-week time point were the lowest for both treatments and regions in comparison with the other periods evaluated.

Notably, intact control samples presented a significant difference in 5mC distribution when comparing regions, being lower in anterior than in posterior segments (*p* = 0.0003). Moreover, the intact posterior control group exhibited the highest amount of 5mC among all the groups, being significantly higher than its respective treated group (*p* < 0.0001) and the control groups after 2 and 4 weeks of regeneration (*p* < 0.0001 and *p* = 0.0002, respectively). It is important to highlight that decitabine treatment successfully affected the incidence of 5mC during the period closest to exposure, with levels subsequently being restored. Interestingly, it appeared reduced again—when compared to the control—by the 4th week only in the posterior treated group.

Curiously, the distribution of 5hmC follows a similar pattern to the one exhibited by 5mC: in contrast with controls, 5hmC was reduced in decitabine-treated animals at all time points and in both regions evaluated, except for the 2-week mark in posterior segments. However, an important distinction is that the levels of 5hmC are generally higher than 5mC, mainly in the intact groups, as well as in the 2-week anterior group, both in control and decitabine treatments (Figure 3B). As previously described in Figure 3A for the case of 5mC, the exposure to decitabine was also linked to reduced 5hmC when compared to the respective controls of the intact anterior and posterior groups, 2-week anterior, and 4-week posterior. Overall, no significant differences between treatments were detected for 5hmC under the conditions tested.

### 3.4. Activity and Gene Expression of Epigenetic Regulatory Enzymes

**Figure 4:**
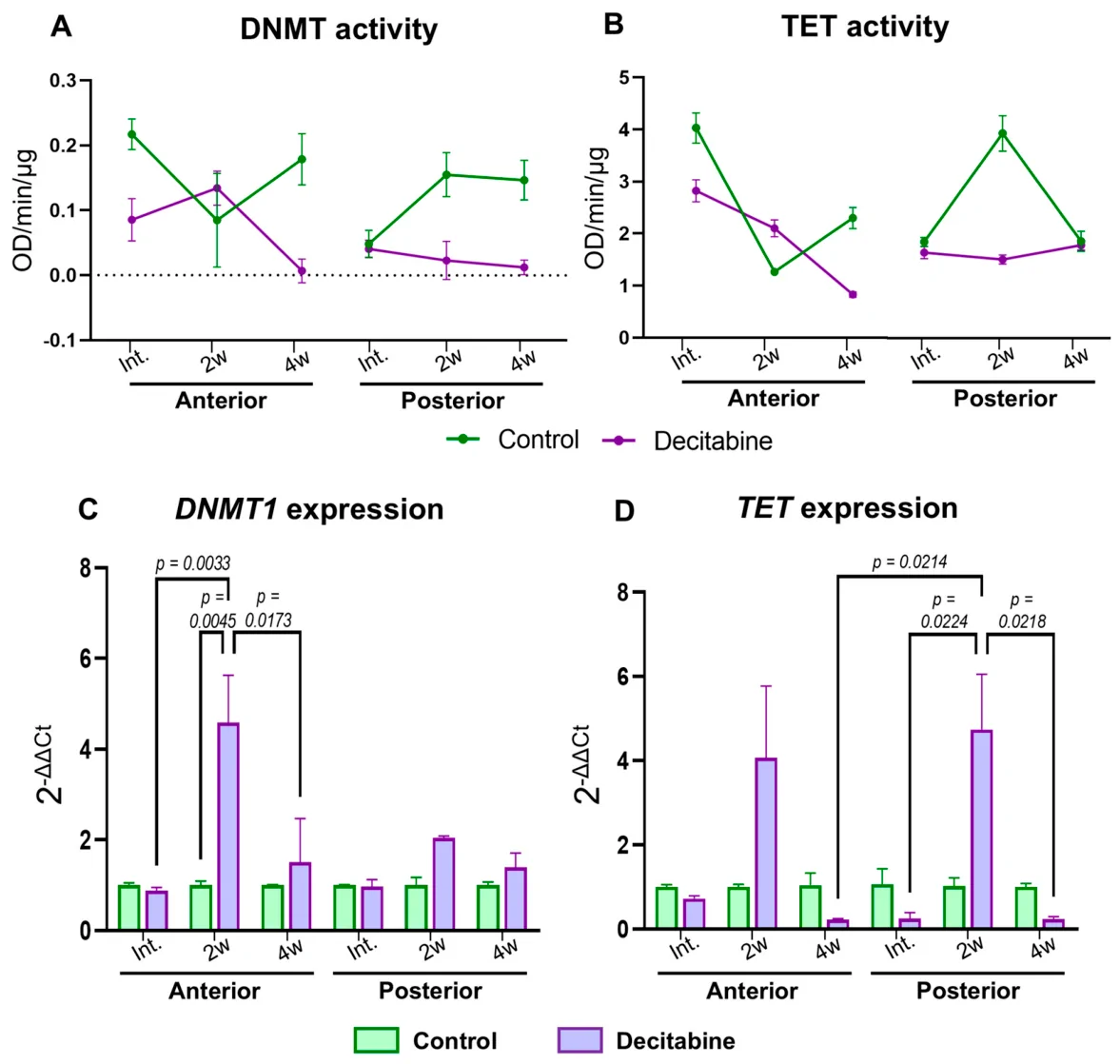
Enzymatic activity of DNA methyltransferase (DNMT, (**A**)) and ten-eleven translocation (TET, (**B**)) by immunocolorimetric assay in intact and regenerated *E. andrei* segments, under treatments and at distinct time points (represented in OD/min/µg). *n* = 6; mean ± SEM. Statistically significant results are described in the results and summarized in Table S2 (please consult the Supplementary Materials). Gene expression of *DNMT1* (**C**) and *TET* (**D**) through qPCR, applying *RPL17* as housekeeping gene. Fold changes in expression are represented as 2^−ΔΔCt^. *n* = 4–6; mean + SEM. All the results (**A**–**D**) show the distinct time points (intact, 2, and 4 weeks post-amputation) and regions (anterior and posterior) evaluated.

The activity of DNMT enzymes in *E. andrei* tissues was reduced after exposure to decitabine compared to controls. This effect could be observed at all the time points (intact, 2-, and 4-week) of both regions examined (anterior and posterior), except for the 2-week anterior blastemas, which exhibited enhanced DNMT activity in contrast to the control (Figure 4A). Anterior intact control groups had a statistically significant increase in DNMT activity compared to the decitabine-exposed group at the 4-week mark, in both anterior (*p* = 0.0188) and posterior (*p* = 0.0256) segments. A similar pattern characterized the effect of decitabine on the activity of ten-eleven translocation (TET) enzymes. However, it is worth noting that even in controls, DNMT enzymes had notably lower activity compared to TET enzymes, and even after exposure to the hypomethylating agent, the latter still evidenced higher activity (Figure 4B). The intact anterior control group presented the most statistically significant (*p* < 0.0001) results compared to all the decitabine-treated groups, at all time points and regions (Table S2). Curiously, the lower values of enzymatic activity for either target did not occur at the intact time point, which was closer to drug exposure; in both cases (DNMT and TET), the lowest activity was observed in the 4-week anterior blastema.

*DNMT1* expression appears slightly reduced right after decitabine exposure (intact groups) in both anterior and posterior segments. Interestingly, its expression pattern showed significant enrichment in the anterior group by the second week of regeneration compared to its control (*p* = 0.0045). This increase in *DNMT1* gene expression by the second week post-amputation is also statistically significant when compared to the other anterior segments in the decitabine-exposed groups (intact with *p* = 0.0033 and 4-week with *p* = 0.0173). Regarding the expression of the *TET* gene, it is coincidentally also elevated in the second week in anterior samples treated with decitabine, when compared to the respective control. However, an elevation in *TET* expression by the 2-week regeneration of decitabine-treated posterior segments is significant in comparison to other treated groups: anterior 4-week (*p* = 0.0214), posterior intact (*p* = 0.0224), and posterior 4-week (*p* = 0.0218) (Figure 4D).

## 4. Discussion

Throughout the centuries, the observation of different organisms and their evolution led to the hypothesis of a broad spectrum of regenerative abilities, which are, to this day, a matter of discussion and questioning among researchers [41]. The highly variable capacity to restore body parts when comparing distinct genera or even species is frequently associated with the evolution of the individual and its surrounding environment [8,42]. As suggested by August Weismann at the end of the 19th century, regeneration is an adaptive trait dependent on multiple factors, such as the frequency of damage, the importance of the organ or appendage for survival, and its complexity [8,41].

Within the animal kingdom, annelids are a very good example of regenerative abilities, being able to restore different body regions, including neuronal and muscular tissues [10,29,43]. The remarkable regenerative capacity of different annelid species has recently made them the subject of multiple studies; for example, in the characterization of morphological features during tissue regeneration [43], as well as the search for specific gene expression profiles [29], associations of regeneration with developmental stages [26], immunity [31], and exposure to different compounds [35].

Previous studies have described the kinetics of wound healing and segment restoration in annelids and specifically earthworms. As depicted by Bodó and collaborators (2021) [31], after amputation, the wound rapidly closes and is covered by undifferentiated cells named epiblasts. The blastema is then formed by proliferating cells located around the injury site, including myoblasts, endoblasts, and mesodermal cells. Until the fourth week of regeneration, the blastema differentiates and assumes the function of the lost segments [31]. As in other organisms, features of the wound, site of injury, and positional cues play a crucial role in the development and patterning of organs and segments of annelids. In this case, the site of injury, considering anterior and posterior regions, directly affects the regeneration process and its aftermath. To exemplify, studies have been conducted to compare the segment restoration capacity of distinct earthworm species in anterior and posterior body sites [9,10,29,43]. In general, anterior blastemas are smaller than the ones formed in the posterior region, and the number of segments removed determines the speed of anterior regeneration.

According to our current observations, *E. andrei* earthworms can restore both anterior and posterior segments upon injury. For example, two weeks after anterior amputation, most animals present with 2 to 3 segments with a width similar to the removed segments, still lacking pigment. In contrast, at the same time point, the posterior blastema has notably thinner segments than the amputated ones or the anterior blastema. The same phenomenon is observed in both control and treated groups, indicating that, in a macroscopic approach, exposure to the hypomethylating agent decitabine does not alter the rate of blastema formation (Figure 1). From another perspective, the presence of the clitellum and the sexual maturation of the annelid worms also interfere with the success of anterior blastema formation [10,43]. The presence or proximity of more complex structures, such as nerve ganglia, digestive tract, and circulatory system, makes the restoration of anterior segments a very delicate process, but also crucial to reestablish feeding as a physiological function necessary for survival and perpetuation of the species. Nevertheless, the exact reason behind the differences in regenerative capacity between organisms remains an underlying question in regenerative biology.

In view of the proposed role of epigenetic mechanisms—especially DNA methylation—in regenerative mechanisms, it raises the possibility of using hypomethylating agents, such as decitabine, to investigate these processes. After cellular uptake, decitabine is sequentially phosphorylated and forms 5-aza-dCTP as a deoxyribonucleoside analog, which is then incorporated into the DNA during the S-phase of the cell cycle, replacing cytosine. This leads to the formation of adducts between DNA and DNMT1, which are later degraded by proteasomes. In this case, the cell cycle resumes, but in the absence of DNMT1, the daughter DNA strands lack the methylation pattern from the paternal strands [32,44]. Through this mechanism, low-dose decitabine causes passive DNA demethylation, altering the genetic landscape and potentially reactivating previously silenced genes.

Because decitabine inhibits DNMT1, which performs mainly maintenance methylation (hemimethylation during cell division), a global reduction in 5mC was expected. The depletion of 5mC marks was seen through immunostaining and dot blot in all decitabine-treated samples when compared to controls, but with statistical significance limited to the posterior intact decitabine group (Figure 3). However, the persistence of this cytosine modification in certain tissues (mainly the coelomic cavity) and its concomitant reduction in other tissues is in accordance with the effect of the drug in tissues with higher cell turnover, once maintenance DNA methylation would be the main mechanism keeping methylation levels in such cases, and the requirement of cells in the S phase of the cell cycle to properly incorporate the drug [45]. In tissues with lower cell turnover (coelomocytes in the coelomic cavity), de novo methylation enzymes (DNMT3a/b) would play a more significant role in forming 5mC, and decitabine would have a less prominent effect. Passive demethylation, when methylation tags in the DNA are diluted during cell divisions and not replenished by maintenance methylation, is one of the mechanisms of action described for decitabine and its analogs [46,47]. Besides the variable effect of the drug depending on the predominant DNMT enzyme in action, variable uptake of the drug depending on tissue and cell type can also be a factor influencing the changes in global 5mC levels.

The variations in both 5mC and 5hmC levels can also be due to the effect of decitabine on the activity of TET enzymes. An enhanced TET dioxygenase activity would utilize 5mC, converting it into 5hmC, therefore decreasing 5mC and increasing 5hmC levels at certain time points. This complementary pattern was observed in intact and 2-week anterior segments, as well as in the intact posterior group (Figure 2 and Figure 3), while an elevation in TET activity was observed at the 2-week mark (Figure 4). When decitabine is administered at higher doses, most of the DNA cannot be recovered after proteasomal degradation of the adducts, leading to cell death. Although the induction of passive demethylation through DNMT1 inhibition is the most established effect of decitabine, recent studies also suggest its role in triggering the active demethylation pathway by inducing TET enzymes (especially TET2 and TET3) [48,49]. Research has collected and described plenty of evidence of the direct and secondary effects of HMA—mainly decitabine and its analogs—on gene expression in different animal models, investigating different pathways and cellular and molecular processes. Moreover, the regeneration process and the site of injury act as additional variables that interfere with the fluctuation of DNA methylation levels.

A previous study by Bodó and collaborators (2021) [31] reported an increase in cell proliferation in 2-week posterior blastemas when compared to intact segments. Intriguingly, our experiments also revealed remarkable fluctuations in DNA methylation by the second week after posterior amputation. For instance, at this time point, 5mC distribution is decreased in most tissue samples treated with decitabine when compared to controls, while 5hmC appears enhanced in most tissues of the same group. The same pattern between the methylation and hydroxymethylation of cytosine is observed from their quantification in genomic DNA in both control and treated groups of posterior segments in the second week. In this regard, IHC observations (Figure 2, Figure S3 and Figure S4) highlighted tissue- and region-related differences in the distribution of DNA methylation marks, their fluctuation during regeneration, and that they can—to some extent—be affected by exposure to decitabine. Correspondingly, variations in methylation levels across experimental groups were revealed through dot blot, which, even though it is considered a less sensitive method, complemented the view of fluctuating DNA methylation due to treatment, tissue/region, and regeneration period from a global genome perspective. It is important to highlight that the use of whole tissue (either at intact or regenerated stages) for genomic DNA isolation and further dot blot assay might hinder the tissue-specific interpretation of the methylation levels here discussed. However, as aforementioned and depicted in Figure 3, the main comparisons focus on the differences between treatments, regions, and time points, acknowledging that specificities of different tissues cannot be assumed.

The 48-h exposure to decitabine was effective in reducing mainly the distribution of 5mC (and slightly, but not significantly, reducing 5hmC), as demonstrated at the intact time point in the dot blot assay when compared to controls (Figure 3A,B). Nonetheless, a fluctuation of both forms of cytosine modifications throughout the regeneration periods and the amputation regions can be observed. By comparing such events between treated and control animals, the role of localized factors might contribute to the alterations in the epigenetic machinery and to the processes involved in tissue restoration.

Additionally, the activity of DNMT and TET is increased in control 2-week-old posterior segments (Figure 4A,B), while the expression of *DNMT1* is enhanced in the decitabine-treated anterior group, and *TET* expression appears increased in both anterior and posterior decitabine-treated segments at the 2-week mark (Figure 4C,D). Despite not knowing the exact mechanism of action of the investigated methylation forms on earthworms’ gene expression—with either repressive or permissive character—there is an evident increment in cell proliferation by the second week of regeneration [31,36]. Correlating this observation with current findings and the hypotheses suggested by other studies, repressive epigenetic marks might be lost, or permissive epigenetic marks might be gained or maintained throughout the tissue restoration process.

It is crucial to highlight that the varying results among experimental groups obtained by the different approaches presented in this study can be interpreted by accounting for differences in the regulation of cellular and molecular processes. For example, the divergence between the enzymatic activity and gene expression results can be explained by several factors: post-translational regulation of proteins, quicker change in mRNA levels than in proteins, availability of cofactors (which directly interfere with enzymatic activity), and presence of enzymatic activators/inhibitors. It is also worth mentioning that specific genes were evaluated in the qPCR experiments, while the colorimetric assays evaluated the total activity of different forms of the enzymes. The fine-tuned regulation layers that are present under physiological conditions and that can be extremely modified under stress conditions, such as upon injury and during tissue restoration, are likely one of the key features to be considered when merging the observations provided by the experiments here described.

To illustrate this scenario, a recent study evaluated the role of clitellar factors in the regeneration of the earthworm *Eudrilus eugeniae* and the expression of specific signaling molecules. It revealed that clitellar factors improved wound closure and induced the upregulation of HoxD3, Wnt3a, and VEGF, for example, as well as stimulated stemness-regulating proteins, such as p53, TCTP, H2AX, and cleaved caspase-3 [50]. Furthermore, another investigation indicates that the earthworm species *Perionyx excavatus* exhibits apoptosis-induced compensatory proliferation (AICP) during posterior regeneration, as evidenced by the observation of apoptosis signals and the concomitant occurrence of cell proliferation and expression of stemness. Additionally, days after amputation, changes in protein expression suggest alterations in Wnt3 signaling and histone H3 patterns [51]. In a comprehensive study analyzing the transcriptome of *E. eugeniae* during anterior regeneration, the expression of genes associated with the proliferation and migration of stem cells was identified, namely NOTCH, RDX, and thymosin beta, for example. In addition, HDAC2 expression was also observed, being correlated with cellular differentiation [52]. Those findings demonstrate that the cellular processes that culminate in regenerative ability, to different extents, are backed up by several layers of cell signaling and protein expression programs, which are then regulated by gene expression systems that include, but are not limited to, DNA methylation patterns. Overall, our findings support that DNA methylation might play a role in annelid regeneration by coordinating changes in gene expression, influencing enzymatic activity, and the distribution of DNA methylation tags throughout different tissues.

As perceived throughout this study, methods relying heavily on antibody-based techniques are adequate alternatives for global methylation assessment when genomic sequencing techniques are not feasible. In this case, we address the main limitation of this study being the lack of genomic information from the animal model used, *Eisenia andrei*. It did, however, benefit from the available transcriptomic data. With that in mind, we consider this a foundational study of the occurrence, distribution, and alterations of DNA methylation in annelid regeneration, additionally describing the effects of body region, period of regeneration, and the hypomethylating drug decitabine on the fluctuation of such epigenetic marks.

## 5. Conclusions

The investigation of the role played by epigenetic mechanisms during annelid tissue regeneration is a fairly unexplored field. Our experiments have described the occurrence of different DNA methylation patterns in the earthworm *Eisenia andrei*, depicting their fluctuation in distinct time points of the regeneration process, considering separate body sites and evaluating multiple tissues. Applying an approach for epigenetic modulation—the exposure to a hypomethylating drug—has pointed out that other factors, probably linked to specific tissue compositions, are capable of strongly influencing DNA methylation. As a limitation, a gene-specific approach was not applied due to the lack of genomic information available for the organism in question; nevertheless, a global methylation perspective is already an advancement in the regeneration biology of invertebrates. Our findings support that the mechanism of action observed by decitabine in higher animals is also occurring in *E. andrei*. It also clarifies the tissue and body region variability of DNA methylation levels in homeostasis, injury, and blastema formation. These results open the way for several innovative ways to study the correlation between epigenetic mechanisms and regeneration. For instance, variations in gene expression, cell signaling molecules, and different cellular processes are key characteristics that should be taken into consideration for further understanding of the topic.

## Data Availability

The data presented in this study are available in the article or Supplementary Materials.

## Supplementary Materials

The following supporting information can be downloaded at https://www.mdpi.com/article/10.3390/biom16081192/s1, Figure S1. Scheme of the experimental procedures for observing DNA methylation mechanisms during anterior and posterior segment regeneration in *Eisenia andrei* earthworms. Figure S2. In vivo observations of regenerating earthworms at intact stage (A–D), and 2 (E–H) and 4 weeks (I–L) after amputation. Anterior (A,B,E,F,I,J) and posterior (C,D,G,H,K,L) regenerating segments are shown in control (A,C,E,G,I,K) and decitabine-treated (B,D,F,H,J,L) earthworms. Dashed lines indicate the separation between the blastema (regenerated tissue) and the original intact segments. Scale bar: 2 mm. Figure S3. Immunohistochemistry staining targeting 5-methylcytosine (A,C) and 5-hydroxymethylcytosine (B,D) showing the blastema formation 2 weeks post-amputation of anterior (A,C) and posterior (B,D) segments. Dashed lines indicate the boundary between original segments and the newly formed ones (side of the new segments is indicated by *). 10× magnification. Scale bars: 100 µm. Figure S4. Immunohistochemistry staining targeting 5-methylcytosine (A–L) and 5-hydroxymethylcytosine (M–X). A–F and M–R: LBSS and decitabine-treated anterior groups; G–L and S–X: LBSS and decitabine-treated posterior groups. 40× magnification. Scale bars: 20 µm. Y: negative control using mouse anti-FITC IgG primary monoclonal antibody; 20×. Z: negative control omitting primary antibody; 20×. Scale bars: 20 µm. Target localization is indicated by: solid arrows in the coelomic cavity; dashed arrows in the gut; arrowheads in the muscle; line arrows in the skin. Table S1: Primers designed for qPCR targeting *DNMT1*, *TET*, and *RPL17*. Table S2: Summary of statistically significant results of the enzymatic activity assays quantifying DNMT and TET activity between treatments and time points. Analysis based on one-way ANOVA and Bonferroni multiple comparison test. *n* = 6.

## Abbreviations

The following abbreviations are used in this manuscript:

5mC	5-methylcytosine
5hmC	5-hydroxymethylcytosine
IHC	Immunohistochemistry
DNMT	DNA-methyltransferase
TET	Ten-eleven translocation
SAM	S-adenosylmethionine
HMAs	Hypomethylating Agents
LBSS	*Lumbricus* Balanced Salt Solution
PFA	Paraformaldehyde
BSA	Bovine Serum Albumin
mAbs	Monoclonal antibodies
HRP	Horseradish Peroxidase
DAB	3,3′-diaminobenzidine
BCA	Bicinchoninic Acid
ANOVA	Analysis of Variance
HoxD3	Homeobox D3
VEGF	Vascular Endothelial Growth Factor
TCTP	Translationally Controlled Tumor Protein
HDAC	Histone Deacetylase
AICP	Apoptosis-Induced Compensatory Proliferation
RDX	Radixin
