# Supplementary material for: DNA Methylation Dynamics in Eisenia andrei Regeneration: The Effects of Time, Region, and a Hypomethylating Agent
Source: Biomolecules. 2026 Aug 14;16(8):1192. doi: 10.3390/biom16081192 (PMC13511733; doi:10.3390/biom16081192)
Supplement: Supplementary file 1 [file biomolecules-16-01192-s001.zip › biomolecules-4474715-supplementary.pdf]

SUPPLEMENTARY MATERIAL

DNA METHYLATION DYNAMICS IN *EISENIA ANDREI* REGENERATION: THE EFFECTS OF TIME, REGION, AND A HYPOMETHYLATING AGENT

Chayeen Brotzki da Costa[1], Péter Németh[1], Péter Engelmann[1, *]

[1]Department of Immunology and Biotechnology, Medical School, Clinical Center, University of Pécs, H-7624 Pécs, Hungary

***Corresponding author:**

Department of Immunology and Biotechnology, Medical School, Clinical Center, University of Pécs, Pécs, H-7624, Szigeti u. 12, Hungary. Tel: + 36-72-536-288, Fax: + 36-72-536-289, email: engelmann.peter@pte.hu

Table of contents

MATERIALS AND METHODS 3
Schematic representation of the methodology 3
Primer design 3
RESULTS 5
***In vivo* observations** 5
Summarized results of immunohistochemistry 6
Summarized results of DNMT and TET activity assays 8
REFERENCES 9

MATERIALS AND METHODS

Schematic representation of the methodology

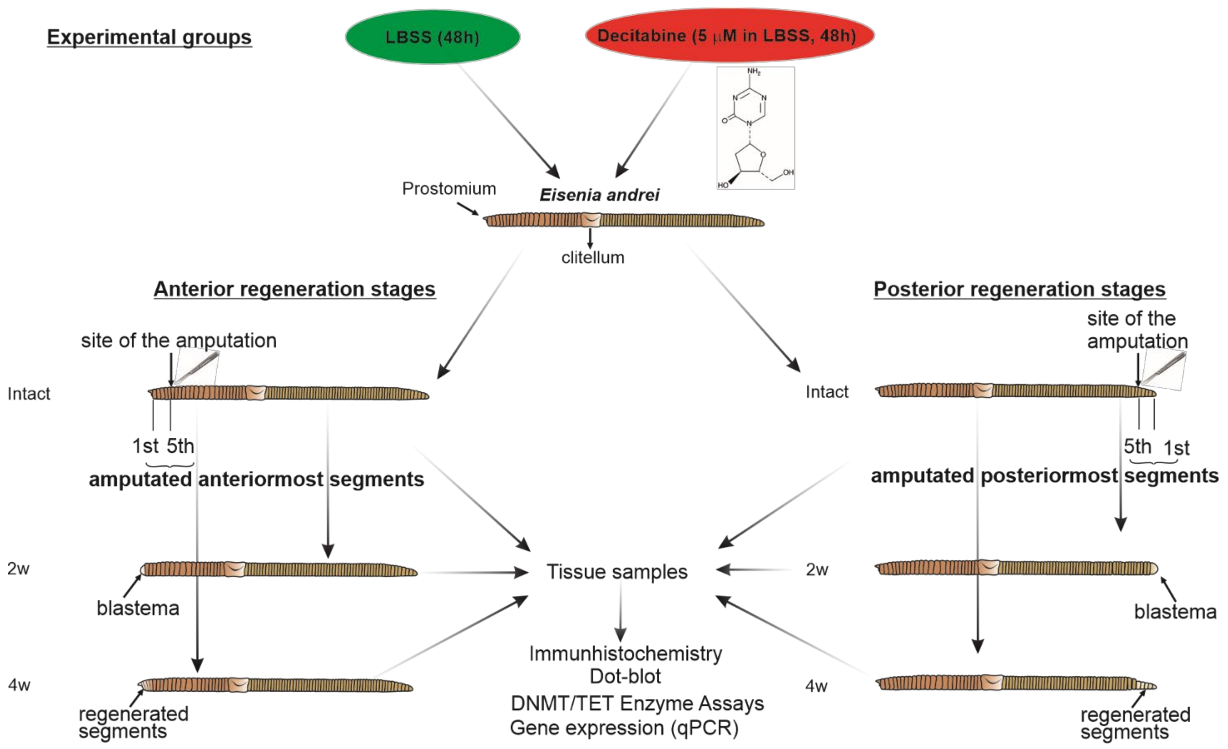

Figure S1. Scheme of the experimental procedures for observing DNA methylation mechanisms during anterior and posterior segment regeneration in *Eisenia andrei* earthworms.

Primer design

To perform quantitative real-time PCR, forward (F) and reverse (R) primers were designed (Integrated DNA Technologies, Coralville, IA, USA) to target the transcripts of *RPL17*, *DNMT1,* and *TET* genes (Table S1), using as reference the sequences of DNMT1 from *Lumbricus terrestris* published by Aigner et al. (2022) [35] and of TET from *Eisenia andrei* published by Shao et al. (2020) [40]. Available partial cDNA sequences were used for multiple alignment with Clustal Omega (version 1.2, University College Dublin, Dublin, Ireland); the most conserved regions within exons were used for primer design with Primer Express (version 3.0, NIH, Bethesda, MD, USA). Primer efficiency was tested using a cDNA concentration range

(10, 100, 1000x dilutions), and melting curve analysis of amplified products was performed at the end of each reaction to confirm the generation of a single PCR product. The housekeeping gene *RPL17* was used for normalization. The next table depicts the primers used and their detailed information. Primer amplification efficiencies were determined from four-point standard curves with linearity values (R^2) ranging from 0.97 to 0.99.

Table S1: Primers designed for qPCR targeting *DNMT1*, *TET*, and *RPL17*.

Primer ID	Sequence (5' – 3')	Efficiency (%)
RPL17 F	GCA GAA TTC AAG GGA CTG GA	102.8
RPL17 R	CTC CTT CTC GGA CAG GAT GA	
Ea-DNMT1 F	CCT CAG ACA AGA AGG AGG GCT AT	93.2
Ea-DNMT1 R	TGC ATT GCT CGT GGT GAG A	
Ea-TET F	AAT CAA CAC TGA AAA TGA GCA TCT CT	90.1
Ea-TET R	ACG CTT GGC CAC CTC AAA	

RESULTS

In vivo observations

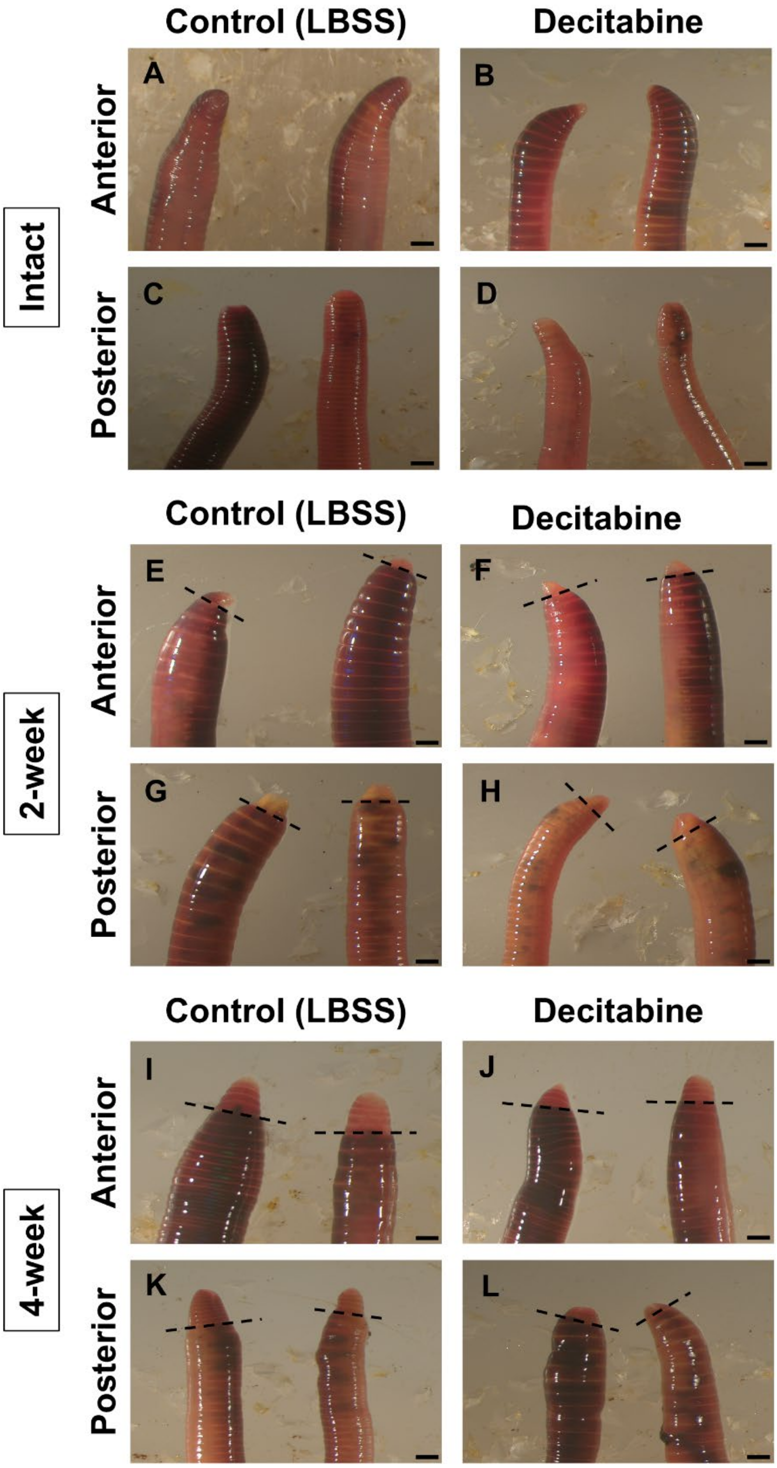

Figure S2. *In vivo* observations of regenerating earthworms at intact stage (A-D), and 2 (E-H) and 4 weeks (I-L) after amputation. Anterior (A, B, E, F, I, J) and posterior (C, D; G, H; K, L) regenerating segments are shown in control (A, C, E, G, I, K) and decitabine-treated (B, D, F, H, J, L) earthworms. Dashed lines indicate the separation between the blastema (regenerated tissue) and the original intact segments. Scale bar: 2 mm.

Summarized results of immunohistochemistry

Microscopic evaluation of signal intensity and positivity was performed by observing four major tissue areas (skin, muscle, gut, and coelomic cavity) from the tissue sections of intact (untreated/treated) and regenerating (untreated/treated) earthworms (Fig. S3, S4).

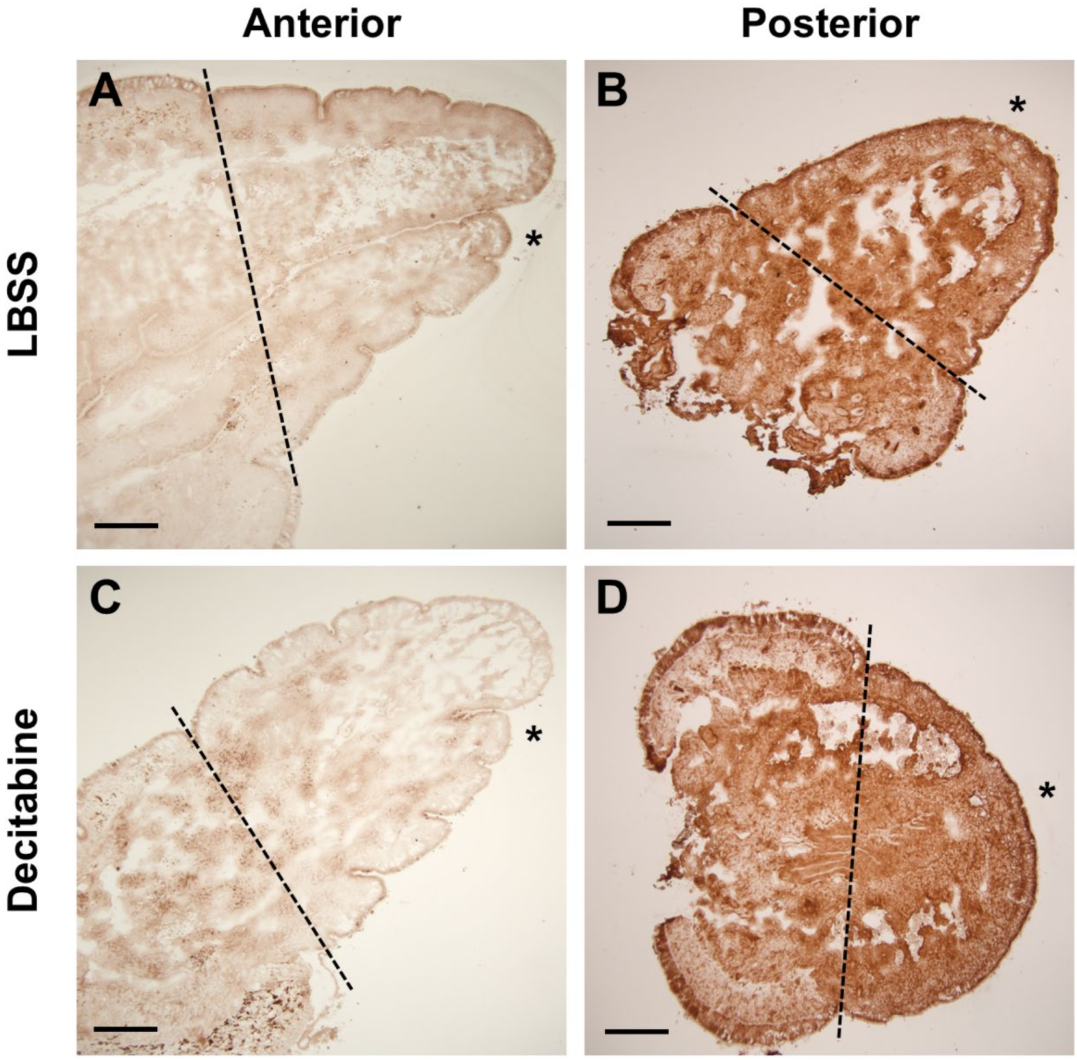

Figure S3. Immunohistochemistry staining targeting 5-methylcytosine (A and C) and 5-hydroxymethylcytosine (B and D) showing the blastema formation 2 weeks post-amputation of anterior (A and C) and posterior (B and D) segments. Dashed lines indicate the boundary between original segments and the newly formed ones (side of the new segments is indicated by *). 10x magnification. Scale bars: 100 μm.

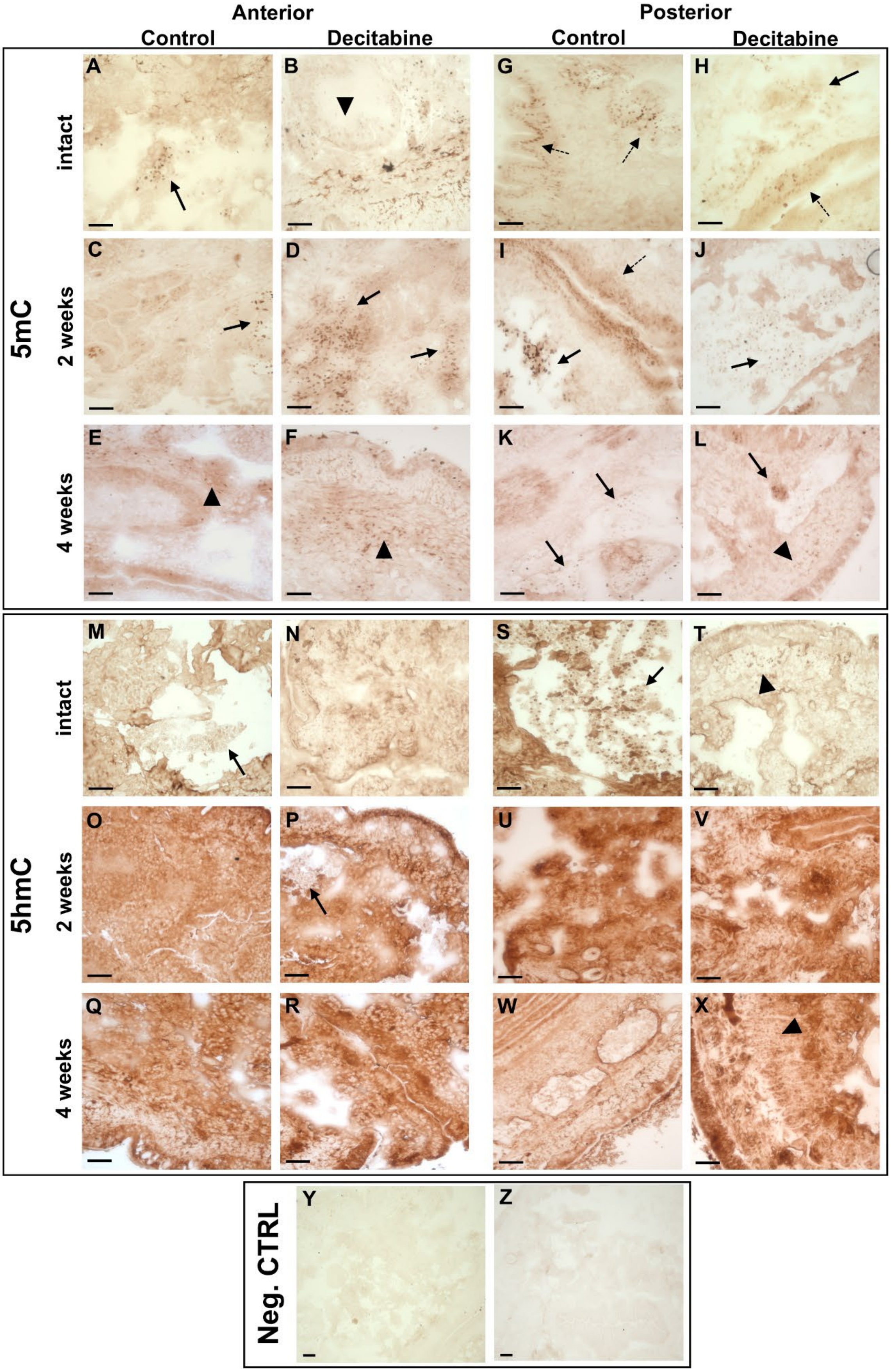

Figure S4. Immunohistochemistry staining targeting 5-methylcytosine (A-L) and 5-hydroxymethylcytosine (M-X). A-F and M-R: LBSS and decitabine-treated anterior groups; G-

L and S-X: LBSS and decitabine-treated posterior groups. 40x magnification. Scale bars: 20 µm. Y: negative control using mouse anti-FITC IgG primary monoclonal antibody; 20x. Z: negative control omitting primary antibody; 20x. Scale bars: 20 µm. Target localization is indicated by: solid arrows in the coelomic cavity; dashed arrows in the gut; arrowheads in the muscle; line arrows in the skin.

Summarized results of DNMT and TET activity assays

Table S2: Summary of statistically significant results of the enzymatic activity assays quantifying DNMT and TET activity between treatments and time points. Analysis based on one-way ANOVA and Bonferroni multiple comparison test. n=6.

**Statistical significance (*p*)**	**DNMT activity**	
0.0188	Control anterior intact	Decitabine anterior 4 weeks
0.0256	Control anterior intact	Decitabine posterior 4 weeks
	**TET activity**	
0.0055	Control anterior intact	Decitabine anterior intact
<0.0001	Control anterior intact	Decitabine posterior intact
<0.0001	Control anterior intact	Decitabine anterior 2 weeks
<0.0001	Control anterior intact	Decitabine posterior 2 weeks
<0.0001	Control anterior intact	Decitabine anterior 4 weeks
<0.0001	Control anterior intact	Decitabine posterior 4 weeks
0.0363	Control posterior intact	Decitabine anterior 4 weeks
0.0002	Decitabine anterior intact	Control anterior 2 weeks
0.0148	Decitabine anterior intact	Control posterior 2 weeks
<0.0001	Decitabine posterior intact	Control posterior 2 weeks
<0.0001	Control posterior 2 weeks	Decitabine anterior 2 weeks
<0.0001	Control posterior 2 weeks	Decitabine posterior 2 weeks
<0.0001	Control posterior 2 weeks	Decitabine anterior 4 weeks
<0.0001	Control posterior 2 weeks	Decitabine posterior 4 weeks
0.0004	Control anterior 4 weeks	Decitabine anterior 4 weeks
0.0318	Control posterior 4 weeks	Decitabine anterior 4 weeks

REFERENCES

[35] Aigner, G. P.; Nenning, P.; Fiechtner, B.; Šrut, M.; Höckner, M. DNA Methylation and detoxification in the earthworm *Lumbricus terrestris* exposed to cadmium and the DNA demethylation agent 5-aza-2′-deoxycytidine. *Toxics* **2022**, *10*, 100. doi: 10.3390/toxics10020100

[40] Shao, Y.; Wang, X.B.; Zhang, J.J.; Li, M.L.; Wu, S.S.; Ma, X.Y.; Wang, X.; Zhao, H.F.; Li, Y.; Zhu, H.H.; et al. Genome and single-cell RNA sequencing of the earthworm *Eisenia andrei* identifies cellular mechanisms underlying regeneration. *Nature Comm* **2020** *11*, 256. doi: 10.1038/s41467-020-16454-8
